# A Novel Homozygous Mutation in *FOXC1* Causes Axenfeld Rieger Syndrome with Congenital Glaucoma

**DOI:** 10.1371/journal.pone.0160016

**Published:** 2016-07-27

**Authors:** Shazia Micheal, Sorath Noorani Siddiqui, Saemah Nuzhat Zafar, Cristina Villanueva-Mendoza, Vianney Cortés-González, Muhammad Imran Khan, Anneke I. den Hollander

**Affiliations:** 1 Department of Ophthalmology, Donders Institute for Brain, Cognition and Behaviour, Radboud University Medical Center, Nijmegen, the Netherlands; 2 Department of Pediatric Ophthalmology, Al-Shifa Eye Trust Hospital, Jhelum Road, Rawalpindi, Pakistan; 3 Genetics Department Asociación Para Evitar la Ceguera en México, Coyoacán, Mexico; 4 Department of Human Genetics, Donders Institute for Brain, Cognition and Behaviour, Radboud University Medical Center, Nijmegen, the Netherlands; University of Iowa, UNITED STATES

## Abstract

**Background:**

Anterior segment dysgenesis (ASD) disorders are a group of clinically and genetically heterogeneous phenotypes in which frequently cornea, iris, and lens are affected. This study aimed to identify novel mutations in *PAX6*, *PITX2* and *FOXC1* in families with anterior segment dysgenesis disorders.

**Methods:**

We studied 14 Pakistani and one Mexican family with Axenfeld Rieger syndrome (ARS; n = 10) or aniridia (n = 5). All affected and unaffected family members underwent full ophthalmologic and general examinations. Total genomic DNA was isolated from peripheral blood. PCR and Sanger sequencing were performed for the exons and intron-exon boundaries of the *FOXC1*, *PAX6*, and *PITX2* genes.

**Results:**

Mutations were identified in five of the 15 probands; four variants were novel and one variant was described previously. A novel *de novo* variant (c.225C>A; p.Tyr75*) was identified in the *PAX6* gene in two unrelated probands with aniridia. In addition, a known variant (c.649C>T; p.Arg217*) in *PAX6* segregated in a family with aniridia. In the *FOXC1* gene, a novel heterozygous variant (c.454T>C; p.Trp152Arg) segregated with the disease in a Mexican family with ARS. A novel homozygous variant (c.92_100del; p.Ala31_Ala33del) in the *FOXC1* gene segregated in a Pakistani family with ARS and congenital glaucoma.

**Conclusions:**

Our study expands the mutation spectrum of the *PAX6* and *FOXC1* genes in individuals with anterior segment dysgenesis disorders. In addition, our study suggests that *FOXC1* mutations, besides typical autosomal dominant ARS, can also cause ARS with congenital glaucoma through an autosomal recessive inheritance pattern. Our results thus expand the disease spectrum of *FOXC1*, and may lead to a better understanding of the role of *FOXC1* in development.

## Introduction

Anterior segment dysgenesis (ASD) disorders encompass a wide variety of developmental conditions in which multiple tissues such as the cornea, iris, and lens are affected [1]. The anterior segment consists of the anterior chamber and the posterior chamber, which are separated by the iris. In the posterior chamber, the ciliary body produces the aqueous humor which provides essential nutrients to the tissues of the anterior segment [2–4]. The aqueous humor is partially drained by the trabecular meshwork, and Schlemm's canal drainage structures located at the anterior segment angle where the iris and cornea meet. These structures that regulate the aqueous humor flow can be affected in patients with anterior segment dysgenesis, which leads to glaucoma in approximately 50% of the cases. These abnormalities may result from the abnormal differentiation of the mesenchymal cells which are responsible for the cornea, iris, and drainage structures development [2–4].

ASD have been classified into different subtypes: aniridia, Axenfeld-Rieger syndrome (ARS), Peters' anomaly (PA), iridogoniodysgenesis, and posterior embryotoxon. They can be inherited either through autosomal recessive or autosomal dominant modes of inheritance, often with incomplete penetrance. Aniridia is a rare congenital disorder of either partial or complete hypoplasia of the iris, and can be associated with other eye defects such as corneal opacification, cataract, glaucoma, lens dislocation, ciliary body hypoplasia, foveal hypoplasia, strabismus, and nystagmus [5–7]. In about two-third of cases it is inherited as an autosomal dominant trait [8, 5]. ARS is a heterogeneous disorder characterized by a broad range of ocular and systemic abnormalities. Ocular features observed in ARS patients include iris stromal hypoplasia, polycoria, corectopia, iridogoniodysgenesis, posterior embryotoxon and iris strands bridging the iridocorneal angle to the trabecular meshwork. The various systemic features observed in ARS patients include facial dysmorphisms (e.g. hypertelorism, prominent forehead, telecanthus), dental anomalies (e.g. hypodontia, microdontia) and a redundant preumbilical skin [9, 10]. Mutations in the *PAX6*, *PITX2*, and *FOXC1* genes have been associated with aniridia and ARS in an autosomal dominant manner [11–16].

The aim of the current study was to identify the genetic causes of aniridia and ARS in 15 Pakistani and Mexican families.

## Materials and Methods

### Ethics statement

This study was approved by the Institutional Review Board of the Pediatric Glaucoma department of Al-Shifa Eye Trust Hospital, Pakistan and the Genetics department of the Asociación Para Evitar la Ceguera en México, Mexico. Written informed consent that complied with the tenets of the Declaration of Helsinki was obtained from every affected and unaffected individual (or his/her guardian) before they were enrolled into the study. The individuals in this manuscript has given written informed consent (as outlined in PLOS consent form) to publish these case details.

### Subjects

Blood samples were collected from affected and unaffected siblings, and from the parents of 14 Pakistani and one Mexican family with anterior segment dysgenesis disorders (ARS n = 10, aniridia n = 5). Genomic DNA was extracted using AutoPure LS DNA Extractor and PUREGEN reagents (Gentra Systems Inc, Minneapolis, Minnesota, USA).

### Polymerase chain reaction and Sanger Sequencing

All coding exons and intron-exon boundaries of the *PAX6*, *PITX2* and *FOXC1* genes were amplified by standard polymerase chain reaction (PCR) (primer sequences available on request). The PCR products were Sanger sequenced using ABI BigDye chemistry (Applied Biosystems Inc, Foster City, California, USA), and were processed through an automated ABI 3730 Sequencer (Applied Biosystems, Inc. The sequencing results were aligned with the consensus sequences using Vector NTI Advance^™^ 2011 software from Life Technologies/Invitrogen (Bleiswijk, Netherlands), by assembling the sequenced contigs. Variants were named according to the nomenclature recommended by the Human Genomic Variation Society (HGVS). Cosegregation analysis was carried out in available family members. The possible effects of variants were predicted by Polymorphism Phenotyping (PolyPhen-2, http://genetics.bwh.harvard.edu/pph2/) and Sorting Intolerant Form Tolerant (SIFT; http://sift.jcvi.org/), PhyloP, Grantham, and MutationTaster.

## Results

### Identification of *PAX6* mutations in three probands with aniridia

**Fig 1:**
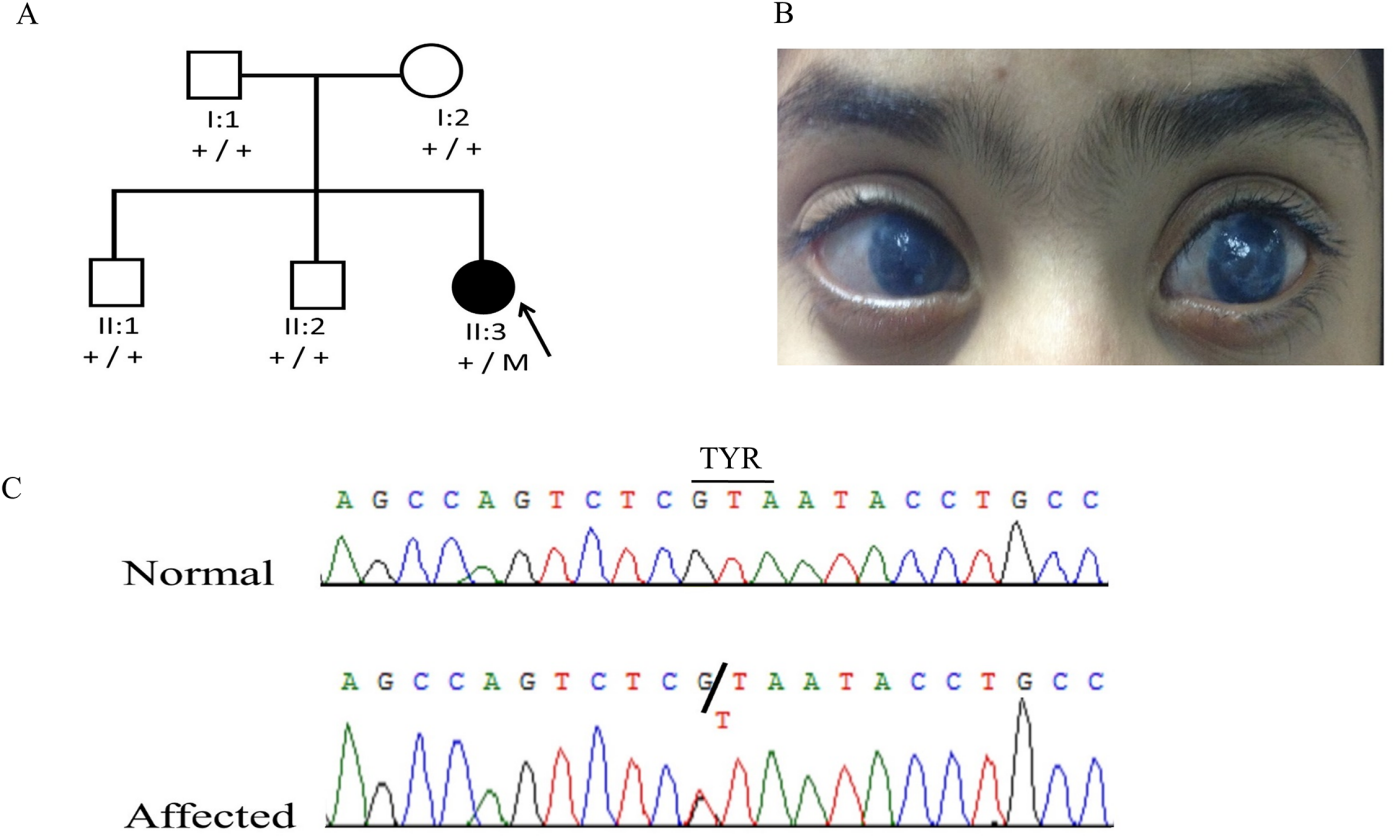
Family 1 from Pakistan with aniridia due to a *de novo* heterozygous *PAX6* mutation. (A) Pedigree and segregation of a novel mutation (c.225C>A; p.Tyr75*) in the *PAX6* gene. (B). Clinical presentation of the affected proband (II:3), corneal neovascularization and opacification in the right and left eyes. (C). DNA sequence chromatogram of *PAX6*.

The proband (II:3) of family 1 (Fig 1) from Pakistan was a 5-year old girl affected with bilateral aniridia, glaucoma, superiorly subluxated lenses, and cataract in the left eye (Fig 1). She underwent bilateral glaucoma surgery and lensectomy. Both parents had normal ocular examinations with 6/6 vision in both eyes. A novel heterozygous nonsense mutation (c.225C>A; p.Tyr75*) in the *PAX6* gene was identified in the proband. The variant was not detected in parents, suggesting that it arose *de novo* in the proband (Fig 1).

The same variation (c.225C>A; p.Tyr75*) was identified in another unrelated 2-year-old girl from Pakistan with bilateral aniridia, corneal opacity and buphthalmos. Her left eye was prosthetic after enucleation. Unfortunately DNA samples of her parents were not available.

**Fig 2:**
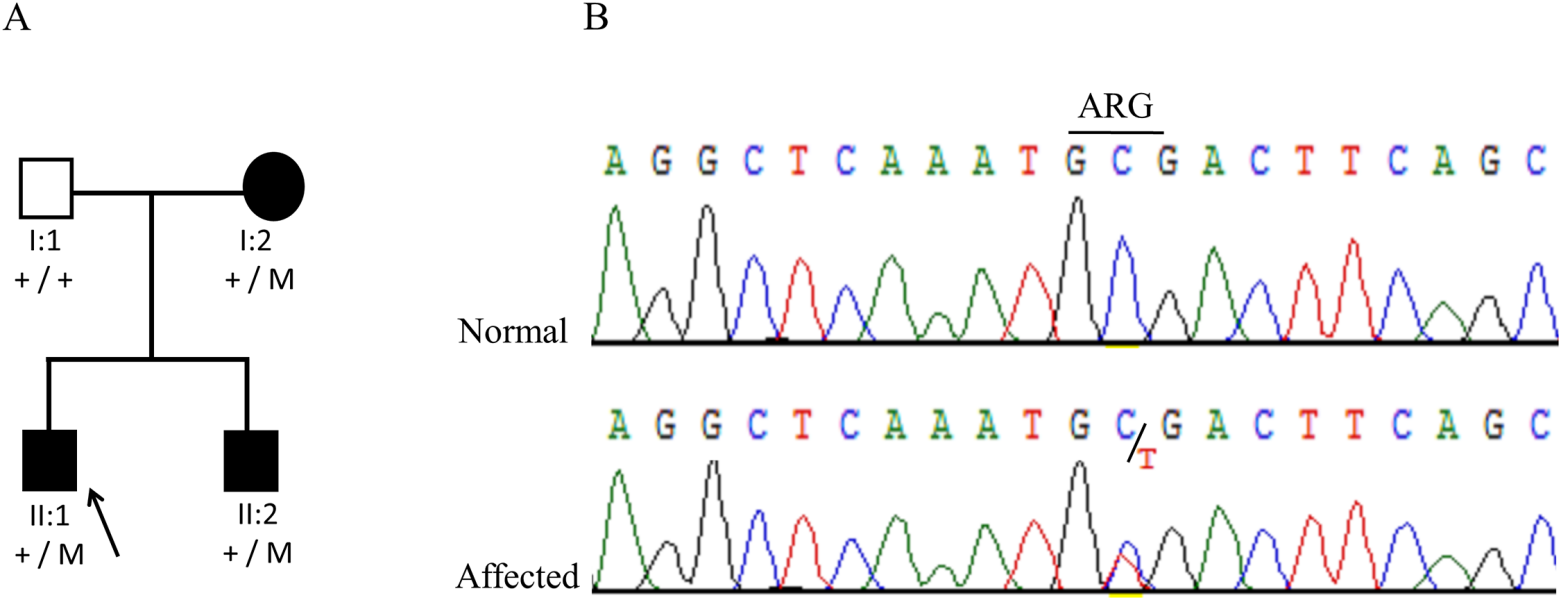
Family 2 from Pakistan with aniridia due to a heterozygous *PAX6* mutation. (a) Pedigree and segregation of a previously described mutation (c.649C>T; p.Arg217*) in the PAX6 gene. (b). DNA sequence chromatogram of *PAX6* for the variant c.649C>T.

Proband II:1 of family 2 (Fig 2) from Pakistan was an 8-year-old boy presenting with aniridia, foveal hypoplasia, aphakia, hazy cornea, cataract and nystagmus. He also had signs of limbal stem cell deficiency and was mentally retarded. His 30-year-old brother (II:2) had similar clinical findings except mental retardation. The mother (I:2) of the proband had no vision in the left eye and had complete aniridia of the right eye. A heterozygous nonsense mutation (c.649C>T; p.Arg217*) in the *PAX6* gene was detected in the proband, and segregated with the disease in the affected mother and brother of the proband (Fig 2).

### Identification of *FOXC1* mutations in two probands with ARS

**Fig 3:**
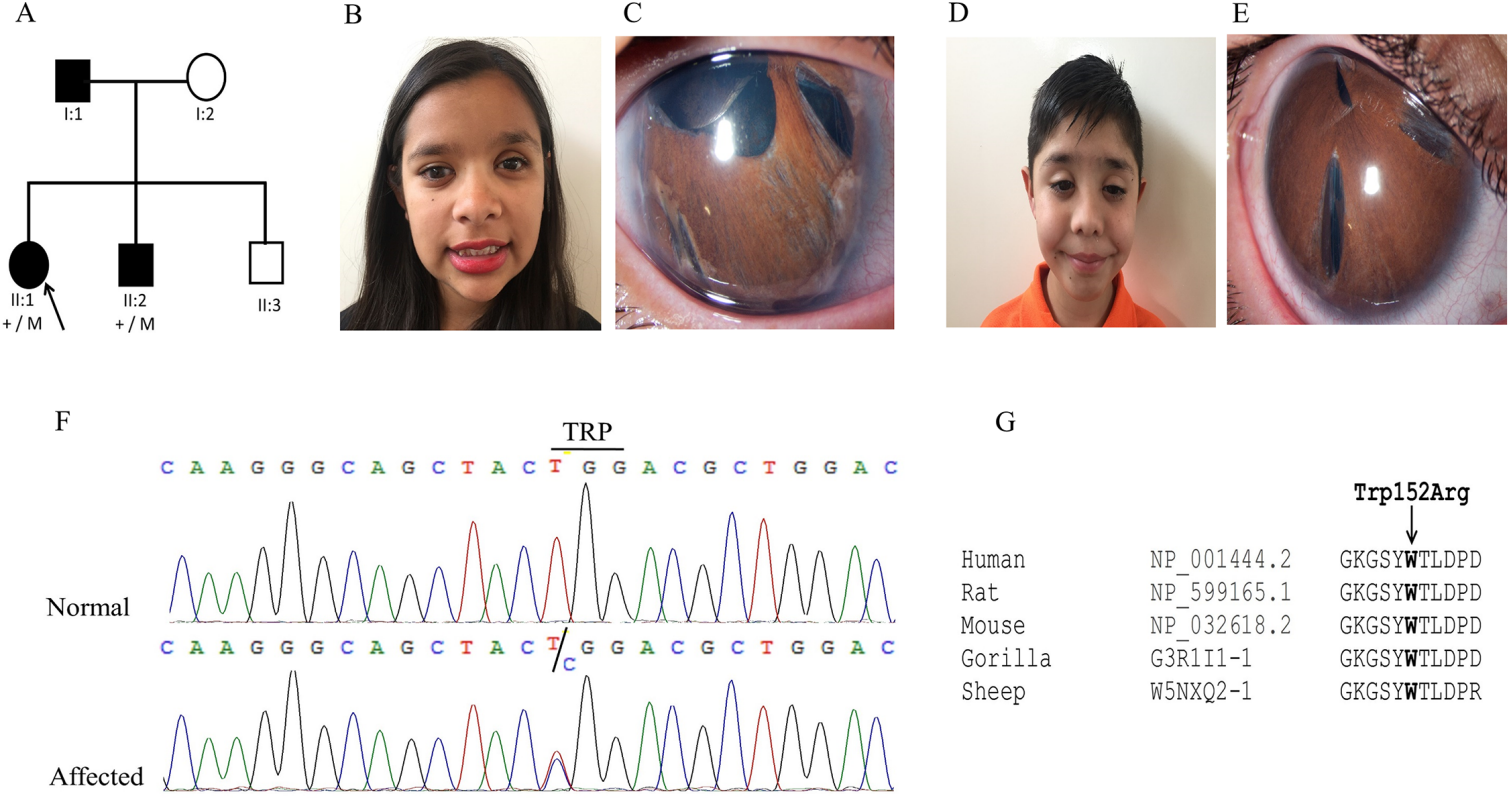
Family 3 from Mexico with ARS due to a heterozygous *FOXC1* mutation. (A) Pedigree and segregation of a novel missense mutation (c.454T>C; p.Trp152Arg) in the *FOXC1* gene. (B). Systemic and ocular characteristics of patient II.1 (C). Slit lamp photograph of right eye with posterior embriotoxon, polycoria, corectopia and iris atrophy. (D). Systemic and ocular characteristics of patient II.2, Midface hypoplasia, thelecantus and broad nasal bridge. (E). Slit lamp photographs of the right eye with posterior embriotoxon, polycoria, corectopia and iris atrophy. (F). Sequence chromatograph of the *FOXC1* variant. (G). Multiple sequence alignment of the region of the FOXC1 protein surrounding the novel p.Trp152Arg mutation in various species. The tryptophan residue (indicated with an arrow) is highly conserved among all species analyzed.

The proband (II:1) of family 3 (Fig 3A) from Mexico was diagnosed with ARS and congenital glaucoma at the age of 6 years. She had a prosthesis of her left eye (Fig 3B and 3C). At the age of 13 years, physical examination showed midface hypoplasia, a flat nose, hypertelorism, telecanthus, enamel hypoplasia and mild deafness. Her 9-year-old brother (II:2) (Fig 3D and 3E) and father (I:1) had similar clinical characteristics. A novel missense variant (c.454T>C; p.Trp152Arg) was identified in the *FOXC1* gene (Fig 3F). The PhyloP score was 4.16 and the Grantham distance was 101. The mutated tryptophan amino acid was conserved among different species (Fig 3G).

**Fig 4:**
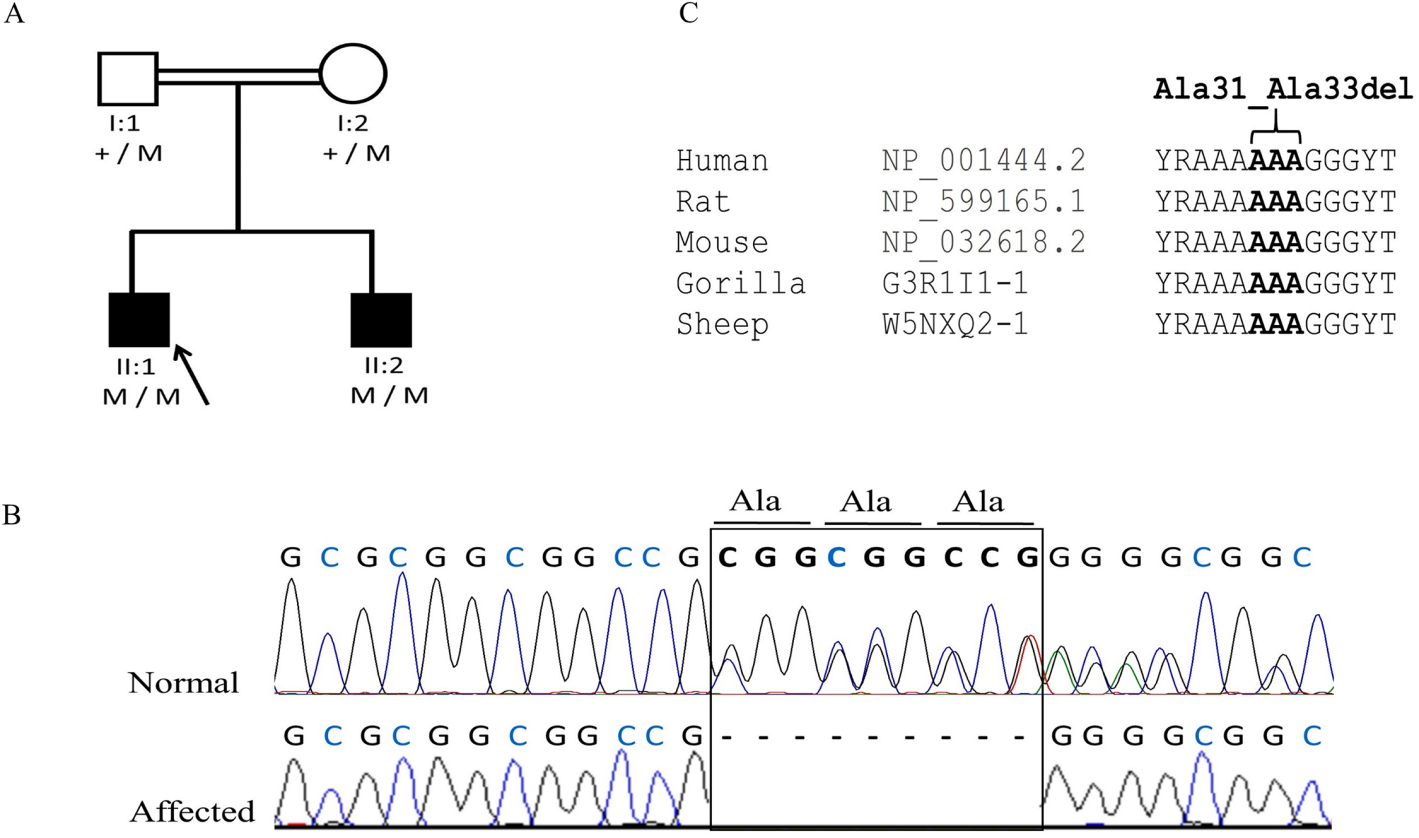
Family 4 from Pakistan with ARS and congenital glaucoma due to a homozygous *FOXC1* mutation. (a) Pedigree and segregation of a novel deletion (c.92_100del; p.Ala31_Ala33del) in the *FOXC1* gene. (b). DNA sequence chromatogram of *FOXC1* demonstrating loss of codons Ala31-33.

Proband II:1 of family 4 (Fig 4A) from Pakistan was a 4-year-old boy with partial aniridia, aphakia, microcornea, cataract, and congenital glaucoma. His 9-year-old brother (II:2) had the similar clinical findings of microcornea, poor vision, and glaucoma. He also had sclerocornea and nystagmus and his left eye was phthisical after surgery for glaucoma. The anterior chamber was not visible due to the corneal opacity. In this family a novel homozygous deletion (c.92_100del; p.Ala31_Ala33del) in the *FOXC1* gene segregated with the disease (Fig 4A and 4B). The mutation leads to an in-frame deletion of three conserved alanine amino acids (Fig 4C).

The variants identified in *PAX6* and *FOXC1* were predicted to be deleterious by SIFT, damaging by Polyphen-2, and disease causing by MutationTaster. In addition the variants were absent in public databases, including the dbSNP132, and Exome Aggregation Consortium (ExAC).

Sanger sequencing of *PITX2* gene did not revealed any variant in all the probands tested.

## Discussion

In the current study we report *PAX 6* mutations in two families and one unrelated proband with aniridia. A novel *de novo* mutation (c.225C>A; p.Tyr75*) was identified in the *PAX6* gene in one family, and the same mutation was identified in an unrelated 2-year old girl with aniridia. In addition, a previously reported mutation (c.649C>T; p.Arg217*) was identified in the *PAX6* gene [17] in another Pakistani family. PAX6 encodes a transcription factor consisting of three domains; a paired domain at the NH_2_-terminus, a homeodomain in the middle, and a transactivation at the COOH-terminus enriched with proline/serine/threonine. Pax6 is widely expressed during normal eye development, [18] however, absence of Pax6 causes anophthalmia in mice [19] and humans [20]. The normal development anterior segment of the eye is dependent on the dose of the Pax6. Therefore, the *PAX6*^*+/−*^ heterozygous null mutations responsible for the haploinsufficiency in humans are characterized by aniridia and other ocular abnormalities due to insufficient dose of Pax6 [14, 11, 21].

The majority (92%) of mutations identified to be associated with aniridia in the *PAX6* gene includes; one or two base pair deletions and insertions resulting a change in the reading frame of the protein, nonsense, and splice site mutations [22–24]. In addition, a minority of mutations are missense mutations (2%), which often cause different phenotypes than null mutations, such as Peters anomaly [25], uveal ectropion [26], ectopia pupillae, cataracts, vascularized cornea, elliptical anterior iris stromal defects and iris hypoplasia. One-third of mutations identified in *PAX6* are *de novo* (http://pax6.hgu.mrc.ac.uk/).

In this study we also identified two novel mutations in the *FOXC1* gene in two families with ARS: a heterozygous missense mutation (c.454T>C; p.Trp152Arg), and a homozygous in-frame deletion (c.92_100del; p.Ala31_Ala33del). In addition to the novel mutations in the *FOXC1* gene segregating with the disease, two known GGC triplet insertions polymorphisms (rs545470261; p.Gly380dup and rs572346201; p.Gly456dup) that lead to a glycine incorporation have been identified in four probands, but none of them were segregating with the disease.

The *FOXC1* gene encodes a member of the forkhead box (FOX) family of transcription factors that are responsible for wide range of important roles such as embryogenesis, tissue-specific gene expression, and tumor development [27]. All forkhead transcription factors contains a highly conserved forkhead DNA-binding domain (FHD). The wing-like structure of the FHD is due to the particular arrangement of the alpha-helixes and beta-sheets. FOXC1 activates the target genes by recognizing and binding to specific DNA sequences in the target genes through the conserved 110-amino-acid FHD [27]. The novel missense mutation (p.Trp152Arg) identified in this study affects a conserved amino acid residue in the second β-strand of the FOXC1 FHD. This mutation affects the same amino acid as the previously reported p.Trp152Gly mutation in a patient with aniridia [28]. The p.Try152Gly mutation has a severe effect on FOXC1 function, affecting its phosphorylation, protein folding, DNA-binding ability, and nuclear localization [28]. The p.Trp152Arg mutation may have a similar consequence on the function of FOXC1. However, it is difficult to explain why there is a difference in the phenotypes of the patients with these mutation as p.Trp152Arg mutation leads to ARS, while the previously reported mutation p.Trp152Gly causes aniridia. Another *FOXC1* mutation (p.Met161Lys) has been reported to cause both aniridia [12] and ARS [13, 29]. The phenotypic variability of overlapping *FOXC1* mutations suggests that perhaps other factors influence the disease outcome.

In this study, we also identified a homozygous in-frame deletion (p.Ala31_Ala33del) of three conserved alanine residues in a family with ARS and congenital glaucoma. These three amino acids are located in the activation domain 1 (AD1) of the FOXC1 protein at the amino terminus, which may affect the binding of FOXC1 with other interactors and may lead to irresponsiveness to ligands that trigger the activation upon binding to the AD1. To date, only heterozygous *FOXC1* mutations have been reported in anterior segment dysgenesis disorders in humans; the family described in this study is the first with a mutation segregating homozygously. Previously, it has been reported that mice with heterozygous (*Foxc1-*/+) and homozygous loss of *Foxc1* (*Foxc1*-/-) have anterior segment abnormalities similar to those reported in humans with ASD and congenital glaucoma, such as iris hypoplasia, small or absence of Schlemm’s canal, severely eccentric pupils, displaced Schwalbe’s line, and aberrantly developed TM [30]. However heterozygous (*Foxc1-*/+) mice had a milder phenotype of ARS compared to the mice with homozygous mutations (*Foxc1*-/-). Homozygous mice exhibit excessive growth of corneal blood and lymphatic vessels which is associated with disorganization of the extracellular matrix (ECM) and increased expression of multiple matrix metalloproteinases [31]. In the current study the patients with the homozygous in-frame deletion of three conserved alanine amino acids were also affected by congenital glaucoma, high IOP (which might be due to the disorganized ECM), pupil corneal vascularization, and mild central corneal opacification at the level of post stroma.

Our study expands the mutation spectrum of the *PAX6* and *FOXC1* genes in individuals with anterior segment dysgenesis disorders. In addition, our study suggests that *FOXC1* mutations, besides typical autosomal dominant ARS, can also cause ARS with congenital glaucoma through an autosomal recessive inheritance pattern. Our results thus expand the disease spectrum of *FOXC1*, and may lead to a better understanding of the role of *FOXC1* in development.

## References

[1] Idrees F, Vaideanu D, Fraser SG, Sowden JC, Khaw PT. A review of anterior segment dysgeneses. Survey of ophthalmology. 2006;51:213–31. 16644364 10.1016/j.survophthal.2006.02.006

[2] Goel M, Picciani RG, Lee RK, Bhattacharya SK. Aqueous humor dynamics: a review. The open ophthalmology journal. 2010;4:52–9. 10.2174/1874364101004010052 21293732 PMC3032230

[3] Tamm ER. The trabecular meshwork outflow pathways: structural and functional aspects. Experimental eye research. 2009;88:648–55. 10.1016/j.exer.2009.02.007 19239914

[4] Weinreb RN, Aung T, Medeiros FA. The pathophysiology and treatment of glaucoma: a review. Jama. 2014;311:1901–11. 10.1001/jama.2014.3192 24825645 PMC4523637

[5] Lee H, Khan R, O'Keefe M. Aniridia: current pathology and management. Acta Ophthalmol. 2008;86:708–15. 10.1111/j.1755-3768.2008.01427.x 18937825

[6] Nelson LB, Spaeth GL, Nowinski TS, Margo CE, Jackson L. Aniridia. A review. Survey of ophthalmology. 1984;28:621–42. 6330922 10.1016/0039-6257(84)90184-x

[7] Hingorani M, Hanson I, van Heyningen V. Aniridia. European journal of human genetics: EJHG. 2012;20:1011–7. 10.1038/ejhg.2012.100 22692063 PMC3449076

[8] Shaw MW, Falls HF, Neel JV. Congenital Aniridia. American journal of human genetics. 1960;12:389–415. 17948455 PMC1932171

[9] Tumer Z, Bach-Holm D. Axenfeld-Rieger syndrome and spectrum of PITX2 and FOXC1 mutations. European journal of human genetics: EJHG. 2009;17:1527–39. 10.1038/ejhg.2009.93 19513095 PMC2987033

[10] Ito YA, Walter MA. Genomics and anterior segment dysgenesis: a review. Clinical & experimental ophthalmology. 2014;42:13–24. 24433355 10.1111/ceo.12152

[11] Jordan T, Hanson I, Zaletayev D, Hodgson S, Prosser J, Seawright A et al The human PAX6 gene is mutated in two patients with aniridia. Nature genetics. 1992;1:328–32. 1302030 10.1038/ng0892-328

[12] Khan AO, Aldahmesh MA, Al-Amri A. Heterozygous FOXC1 mutation (M161K) associated with congenital glaucoma and aniridia in an infant and a milder phenotype in her mother. Ophthalmic genetics. 2008;29:67–71. 10.1080/13816810801908152 18484311

[13] Komatireddy S, Chakrabarti S, Mandal AK, Reddy AB, Sampath S, Panicker SG et al Mutation spectrum of FOXC1 and clinical genetic heterogeneity of Axenfeld-Rieger anomaly in India. Molecular vision. 2003;9:43–8. 12592227

[14] Ton CC, Hirvonen H, Miwa H, Weil MM, Monaghan P, Jordan T et al Positional cloning and characterization of a paired box- and homeobox-containing gene from the aniridia region. Cell. 1991;67:1059–74. 1684738 10.1016/0092-8674(91)90284-6

[15] Yin HF, Fang XY, Jin CF, Yin JF, Li JY, Zhao SJ et al Identification of a novel frameshift mutation in PITX2 gene in a Chinese family with Axenfeld-Rieger syndrome. Journal of Zhejiang University Science B. 2014;15:43–50. 10.1631/jzus.B1300053 24390743 PMC3891117

[16] Yun JW, Cho HK, Oh SY, Ki CS, Kee C. Novel c.300_301delinsT mutation in PITX2 in a Korean family with Axenfeld-Rieger syndrome. Annals of laboratory medicine. 2013;33:360–3. 10.3343/alm.2013.33.5.360 24003428 PMC3756242

[17] Xiao X, Li S, Zhang Q. Microphthalmia, late onset keratitis, and iris coloboma/aniridia in a family with a novel PAX6 mutation. Ophthalmic genetics. 2012;33:119–21. 10.3109/13816810.2011.642452 22171686

[18] Grindley JC, Davidson DR, Hill RE. The role of Pax-6 in eye and nasal development. Development. 1995;121:1433–42. 7789273 10.1242/dev.121.5.1433

[19] Hill RE, Favor J, Hogan BL, Ton CC, Saunders GF, Hanson IM et al Mouse small eye results from mutations in a paired-like homeobox-containing gene. Nature. 1991;354:522–5. 1684639 10.1038/354522a0

[20] Glaser T, Jepeal L, Edwards JG, Young SR, Favor J, RL Maas. PAX6 gene dosage effect in a family with congenital cataracts, aniridia, anophthalmia and central nervous system defects. Nature genetics. 1994;7:463–71. 7951315 10.1038/ng0894-463

[21] Glaser T, Walton DS, Maas RL. Genomic structure, evolutionary conservation and aniridia mutations in the human PAX6 gene. Nature genetics. 1992;2:232–9. 1345175 10.1038/ng1192-232

[22] Prosser J, van Heyningen V. PAX6 mutations reviewed. Human mutation. 1998;11:93–108. 9482572 10.1002/(SICI)1098-1004(1998)11:2<93::AID-HUMU1>3.0.CO;2-M

[23] Tzoulaki I, White IM, Hanson IM. PAX6 mutations: genotype-phenotype correlations. BMC genetics. 2005;6:27 15918896 10.1186/1471-2156-6-27 PMC1156885

[24] Kokotas H, Petersen MB. Clinical and molecular aspects of aniridia. Clinical genetics. 2010;77:409–20. 10.1111/j.1399-0004.2010.01372.x 20132240

[25] Hanson IM, Fletcher JM, Jordan T, Brown A, Taylor D, Adams RJ et al Mutations at the PAX6 locus are found in heterogeneous anterior segment malformations including Peters' anomaly. Nature genetics. 1994;6:168–73. 8162071 10.1038/ng0294-168

[26] Azuma N, Yamada M. Missense mutation at the C terminus of the PAX6 gene in ocular anterior segment anomalies. Investigative ophthalmology & visual science. 1998;39:828–30. 9538891

[27] Lehmann OJ, Sowden JC, Carlsson P, Jordan T, Bhattacharya SS. Fox's in development and disease. Trends in genetics: TIG. 2003;19:339–44. 12801727 10.1016/S0168-9525(03)00111-2

[28] Ito YA, Footz TK, Berry FB, Mirzayans F, Yu M, Khan AO et al Severe molecular defects of a novel FOXC1 W152G mutation result in aniridia. Investigative ophthalmology & visual science. 2009;50:3573–9. 19279310 10.1167/iovs.08-3032

[29] Panicker SG, Sampath S, Mandal AK, Reddy AB, Ahmed N, Hasnain SE. Novel mutation in FOXC1 wing region causing Axenfeld-Rieger anomaly. Investigative ophthalmology & visual science. 2002;43:3613–6. 12454026

[30] Smith RS, Zabaleta A, Kume T, Savinova OV, Kidson SH, Martin JE et al Haploinsufficiency of the transcription factors FOXC1 and FOXC2 results in aberrant ocular development. Human molecular genetics. 2000;9:1021–32. 10767326 10.1093/hmg/9.7.1021

[31] Seo S, Singh HP, Lacal PM, Sasman A, Fatima A, Liu T et al Forkhead box transcription factor FoxC1 preserves corneal transparency by regulating vascular growth. Proceedings of the National Academy of Sciences of the United States of America. 2012;109:2015–20. 10.1073/pnas.1109540109 22171010 PMC3277512

